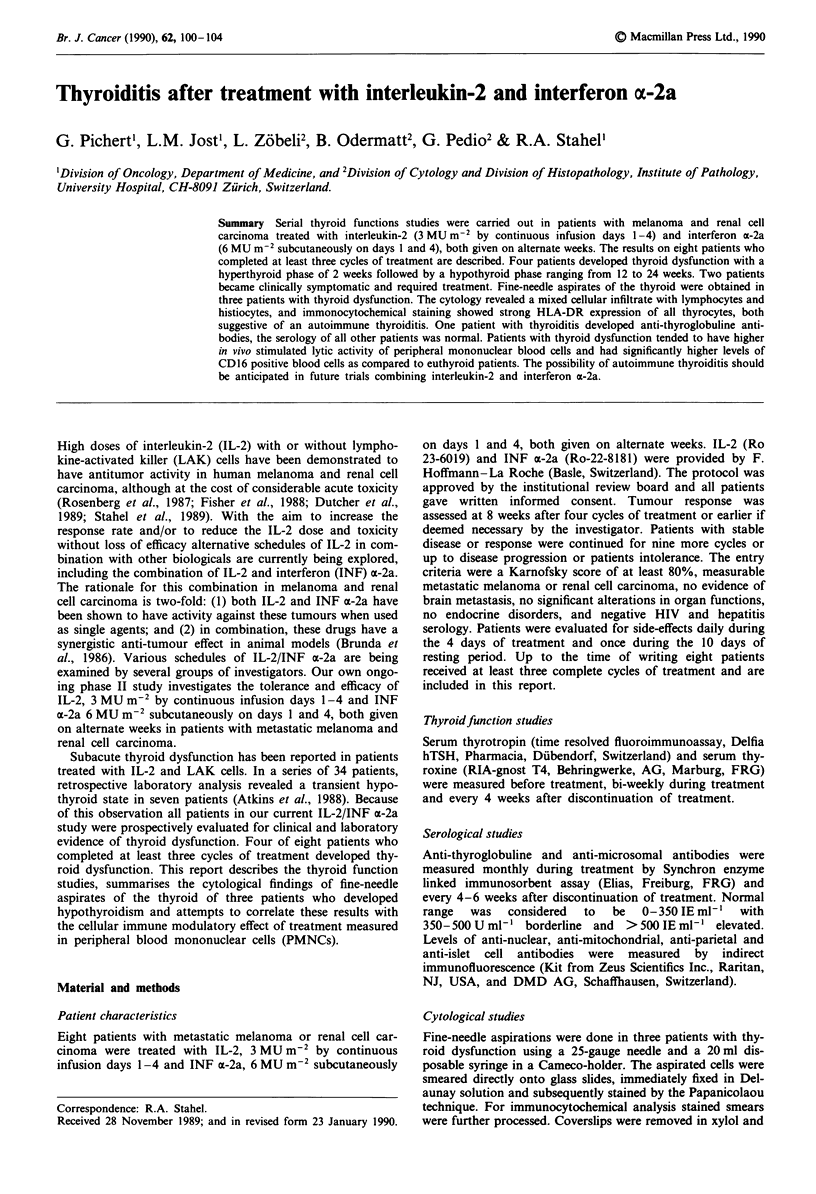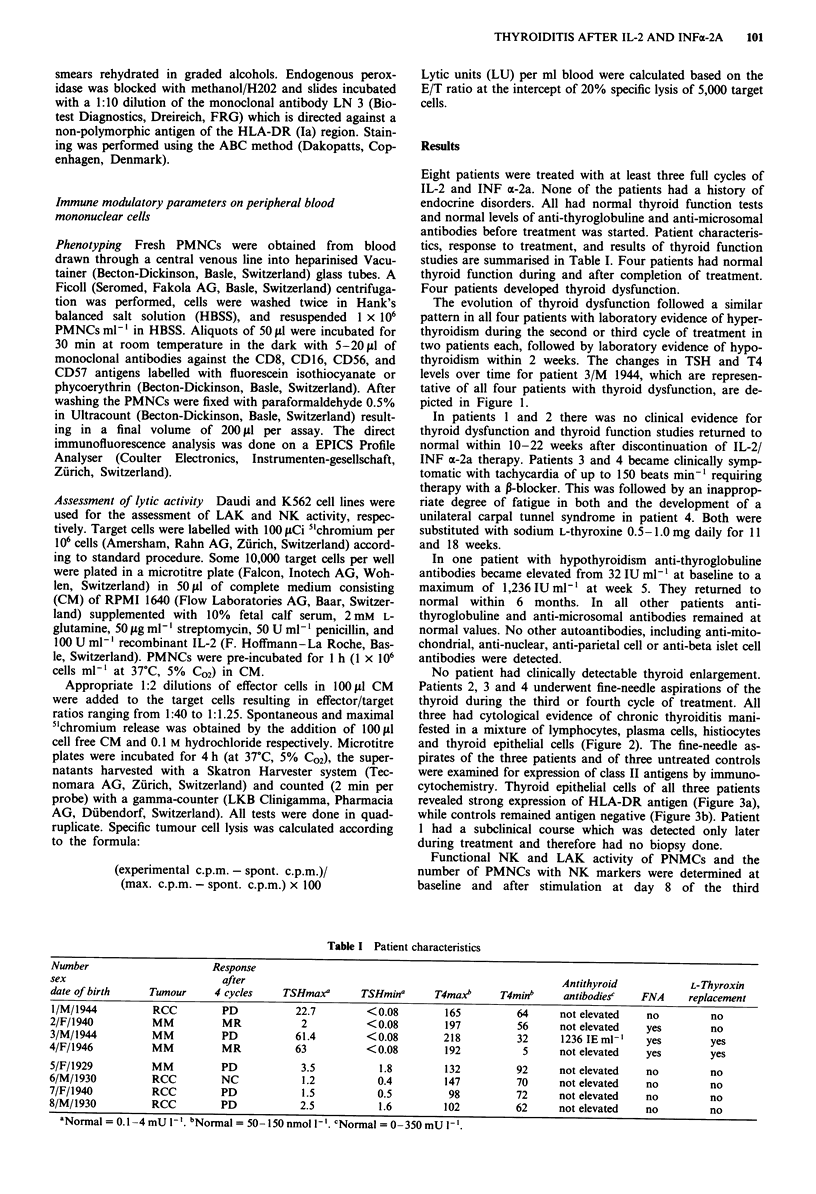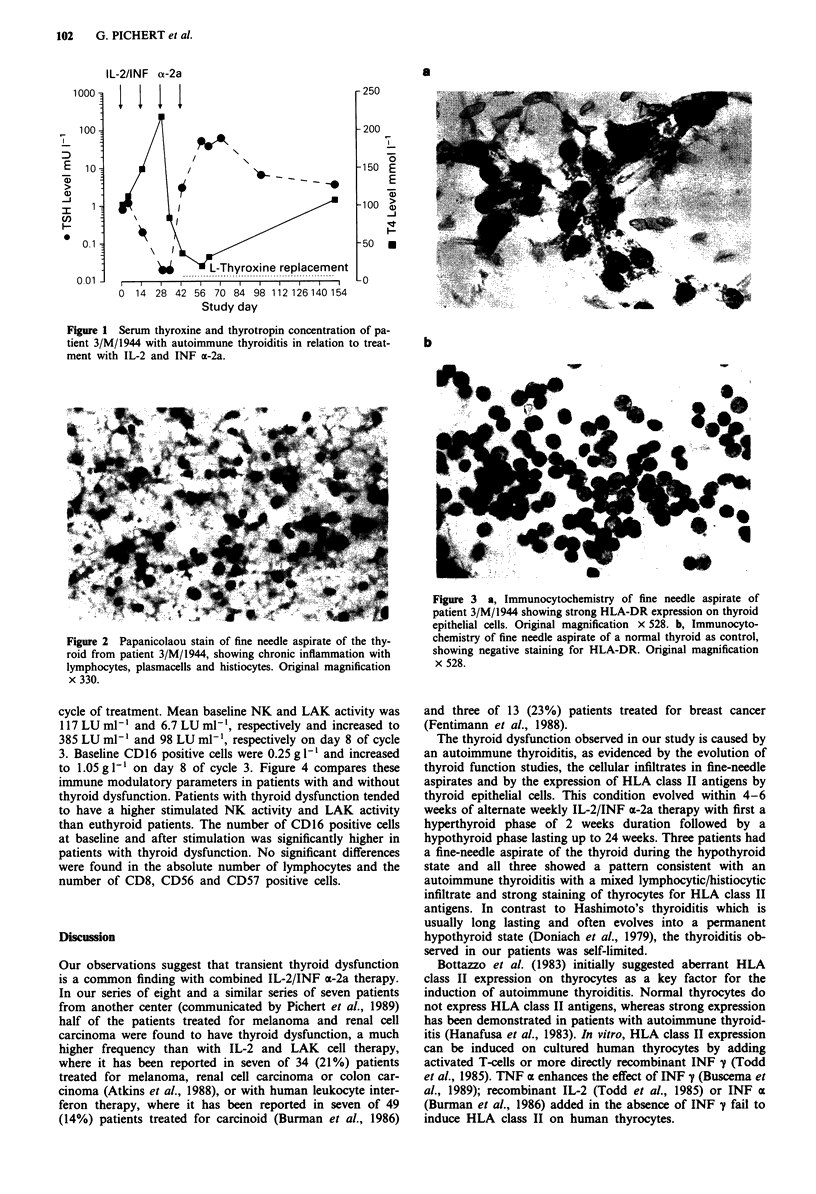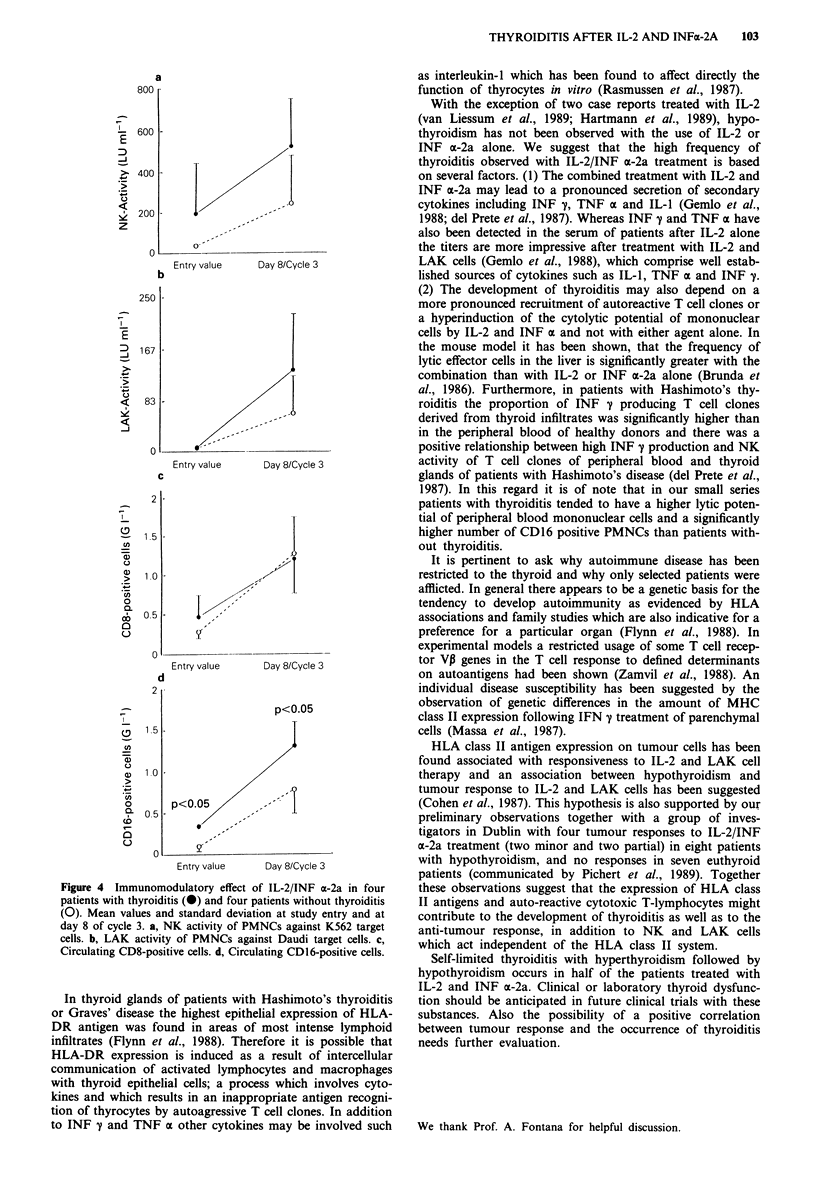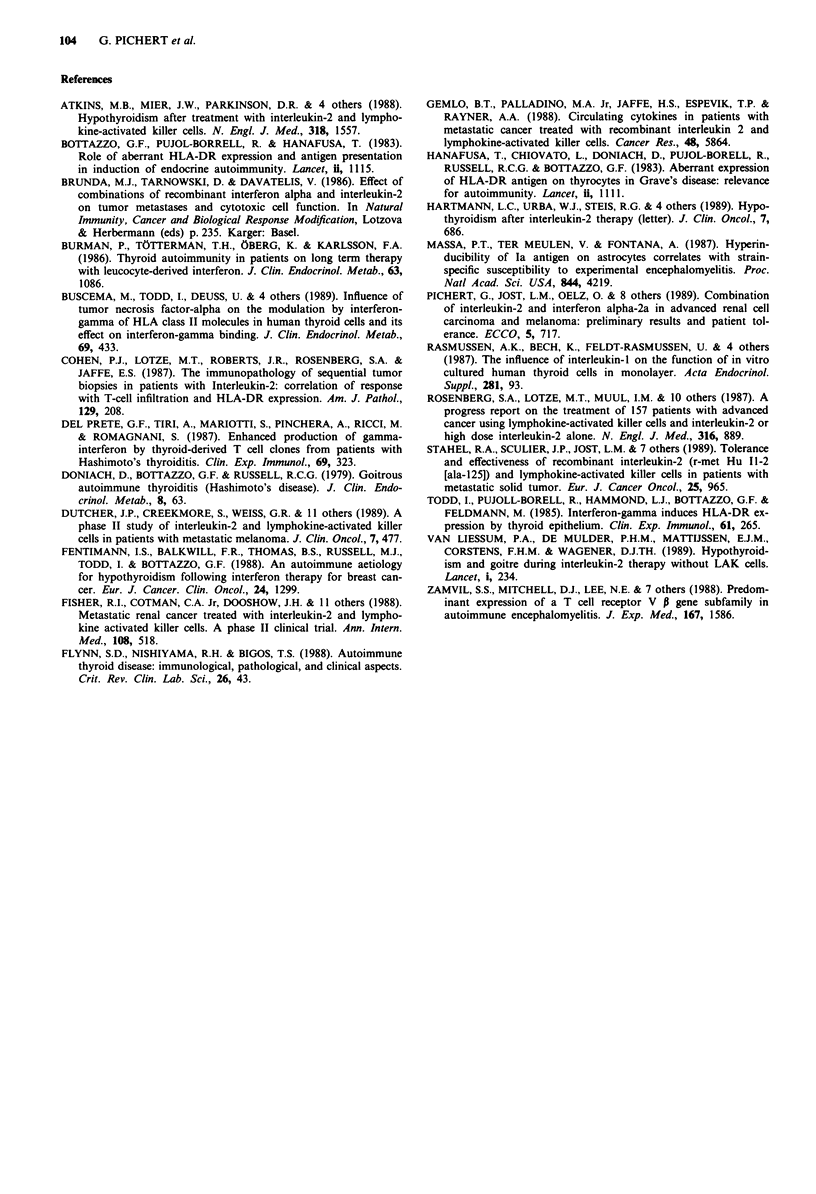# Thyroiditis after treatment with interleukin-2 and interferon alpha-2a.

**DOI:** 10.1038/bjc.1990.237

**Published:** 1990-07

**Authors:** G. Pichert, L. M. Jost, L. Zöbeli, B. Odermatt, G. Pedia, R. A. Stahel

**Affiliations:** Department of Medicine, University Hospital, Zürich, Switzerland.

## Abstract

**Images:**


Br. J. Cancer (1990), 62, 100-104 © Macmillan Press Ltd., 1990

### Thyroiditis after treatment with interleukin-2 and interferon α-2a

G. Pichert[1], L.M. Jost[1], L. Zöbeli[2], B. Odermatt[2], G. Pedio[2] & R.A. Stahel[1]

[1]*Division of Oncology, Department of Medicine, and* [2]*Division of Cytology and Division of Histopathology, Institute of Pathology, University Hospital, CH-8091 Zürich, Switzerland.*

**Summary** Serial thyroid functions studies were carried out in patients with melanoma and renal cell carcinoma treated with interleukin-2 (3 MU $m^{-2}$ by continuous infusion days 1-4) and interferon α-2a (6 MU $m^{-2}$ subcutaneously on days 1 and 4), both given on alternate weeks. The results on eight patients who completed at least three cycles of treatment are described. Four patients developed thyroid dysfunction with a hyperthyroid phase of 2 weeks followed by a hypothyroid phase ranging from 12 to 24 weeks. Two patients became clinically symptomatic and required treatment. Fine-needle aspirates of the thyroid were obtained in three patients with thyroid dysfunction. The cytology revealed a mixed cellular infiltrate with lymphocytes and histiocytes, and immonocytochemical staining showed strong HLA-DR expression of all thyrocytes, both suggestive of an autoimmune thyroiditis. One patient with thyroiditis developed anti-thyroglobuline antibodies, the serology of all other patients was normal. Patients with thyroid dysfunction tended to have higher *in vivo* stimulated lytic activity of peripheral mononuclear blood cells and had significantly higher levels of CD16 positive blood cells as compared to euthyroid patients. The possibility of autoimmune thyroiditis should be anticipated in future trials combining interleukin-2 and interferon α-2a.

High doses of interleukin-2 (IL-2) with or without lymphokine-activated killer (LAK) cells have been demonstrated to have antitumor activity in human melanoma and renal cell carcinoma, although at the cost of considerable acute toxicity (Rosenberg *et al.*, 1987; Fisher *et al.*, 1988; Dutcher *et al.*, 1989; Stahel *et al.*, 1989). With the aim to increase the response rate and/or to reduce the IL-2 dose and toxicity without loss of efficacy alternative schedules of IL-2 in combination with other biologicals are currently being explored, including the combination of IL-2 and interferon (INF) α-2a. The rationale for this combination in melanoma and renal cell carcinoma is two-fold: (1) both IL-2 and INF α-2a have been shown to have activity against these tumours when used as single agents; and (2) in combination, these drugs have a synergistic anti-tumour effect in animal models (Brunda *et al.*, 1986). Various schedules of IL-2/INF α-2a are being examined by several groups of investigators. Our own ongoing phase II study investigates the tolerance and efficacy of IL-2, 3 MU $m^{-2}$ by continuous infusion days 1-4 and INF α-2a 6 MU $m^{-2}$ subcutaneously on days 1 and 4, both given on alternate weeks in patients with metastatic melanoma and renal cell carcinoma.

Subacute thyroid dysfunction has been reported in patients treated with IL-2 and LAK cells. In a series of 34 patients, retrospective laboratory analysis revealed a transient hypothyroid state in seven patients (Atkins *et al.*, 1988). Because of this observation all patients in our current IL-2/INF α-2a study were prospectively evaluated for clinical and laboratory evidence of thyroid dysfunction. Four of eight patients who completed at least three cycles of treatment developed thyroid dysfunction. This report describes the thyroid function studies, summarises the cytological findings of fine-needle aspirates of the thyroid of three patients who developed hypothyroidism and attempts to correlate these results with the cellular immune modulatory effect of treatment measured in peripheral blood mononuclear cells (PMNCs).

### Material and methods

### *Patient characteristics*

Eight patients with metastatic melanoma or renal cell carcinoma were treated with IL-2, 3 MU $m^{-2}$ by continuous infusion days 1-4 and INF α-2a, 6 MU $m^{-2}$ subcutaneously on days 1 and 4, both given on alternate weeks. IL-2 (Ro 23-6019) and INF α-2a (Ro-22-8181) were provided by F. Hoffmann-La Roche (Basle, Switzerland). The protocol was approved by the institutional review board and all patients gave written informed consent. Tumour response was assessed at 8 weeks after four cycles of treatment or earlier if deemed necessary by the investigator. Patients with stable disease or response were continued for nine more cycles or up to disease progression or patients intolerance. The entry criteria were a Karnofsky score of at least 80%, measurable metastatic melanoma or renal cell carcinoma, no evidence of brain metastasis, no significant alterations in organ functions, no endocrine disorders, and negative HIV and hepatitis serology. Patients were evaluated for side-effects daily during the 4 days of treatment and once during the 10 days of resting period. Up to the time of writing eight patients received at least three complete cycles of treatment and are included in this report.

### *Thyroid function studies*

Serum thyrotropin (time resolved fluoroimmunoassay, Delfia hTSH, Pharmacia, Dübendorf, Switzerland) and serum thyroxine (RIA-gnost T4, Behringwerke, AG, Marburg, FRG) were measured before treatment, bi-weekly during treatment and every 4 weeks after discontinuation of treatment.

### *Serological studies*

Anti-thyroglobuline and anti-microsomal antibodies were measured monthly during treatment by Synchron enzyme linked immunosorbent assay (Elias, Freiburg, FRG) and every 4-6 weeks after discontinuation of treatment. Normal range was considered to be 0-350 IE $ml^{-1}$ with 350-500 U $ml^{-1}$ borderline and >500 IE $ml^{-1}$ elevated. Levels of anti-nuclear, anti-mitochondrial, anti-parietal and anti-islet cell antibodies were measured by indirect immunofluorescence (Kit from Zeus Scientifics Inc., Raritan, NJ, USA, and DMD AG, Schaffhausen, Switzerland).

### *Cytological studies*

Fine-needle aspirations were done in three patients with thyroid dysfunction using a 25-gauge needle and a 20 ml disposable syringe in a Cameco-holder. The aspirated cells were smeared directly onto glass slides, immediately fixed in Delaunay solution and subsequently stained by the Papanicolaou technique. For immunocytochemical analysis stained smears were further processed. Coverslips were removed in xylol and

Correspondence: R.A. Stahel.
Received 28 November 1989; and in revised form 23 January 1990.

smears rehydrated in graded alcohols. Endogenous peroxidase was blocked with methanol/H202 and slides incubated with a 1:10 dilution of the monoclonal antibody LN 3 (Biotest Diagnostics, Dreireich, FRG) which is directed against a non-polymorphic antigen of the HLA-DR (Ia) region. Staining was performed using the ABC method (Dakopatts, Copenhagen, Denmark).

*Immune modulatory parameters on peripheral blood mononuclear cells*

*Phenotyping* Fresh PMNCs were obtained from blood drawn through a central venous line into heparinised Vacutainer (Becton-Dickinson, Basle, Switzerland) glass tubes. A Ficoll (Seromed, Fakola AG, Basle, Switzerland) centrifugation was performed, cells were washed twice in Hank's balanced salt solution (HBSS), and resuspended $1 \times 10^6$ PMNCs $ml^{-1}$ in HBSS. Aliquots of 50 μl were incubated for 30 min at room temperature in the dark with 5–20 μl of monoclonal antibodies against the CD8, CD16, CD56, and CD57 antigens labelled with fluorescein isothiocyanate or phycoerythrin (Becton-Dickinson, Basle, Switzerland). After washing the PMNCs were fixed with paraformaldehyde 0.5% in Ultracount (Becton-Dickinson, Basle, Switzerland) resulting in a final volume of 200 μl per assay. The direct immunofluorescence analysis was done on a EPICS Profile Analyser (Coulter Electronics, Instrumenten-gesellschaft, Zürich, Switzerland).

*Assessment of lytic activity* Daudi and K562 cell lines were used for the assessment of LAK and NK activity, respectively. Target cells were labelled with 100 μCi $^{51}$chromium per $10^6$ cells (Amersham, Rahn AG, Zürich, Switzerland) according to standard procedure. Some 10,000 target cells per well were plated in a microtitre plate (Falcon, Inotech AG, Wohlen, Switzerland) in 50 μl of complete medium consisting (CM) of RPMI 1640 (Flow Laboratories AG, Baar, Switzerland) supplemented with 10% fetal calf serum, 2 mM L-glutamine, 50 μg $ml^{-1}$ streptomycin, 50 U $ml^{-1}$ penicillin, and 100 U $ml^{-1}$ recombinant IL-2 (F. Hoffmann-La Roche, Basle, Switzerland). PMNCs were pre-incubated for 1 h ($1 \times 10^6$ cells $ml^{-1}$ at 37°C, 5% $C_{O2}$) in CM.

Appropriate 1:2 dilutions of effector cells in 100 μl CM were added to the target cells resulting in effector/target ratios ranging from 1:40 to 1:1.25. Spontaneous and maximal $^{51}$chromium release was obtained by the addition of 100 μl cell free CM and 0.1 M hydrochloride respectively. Microtitre plates were incubated for 4 h (at 37°C, 5% $C_{O2}$), the supernatants harvested with a Skatron Harvester system (Tecnomara AG, Zürich, Switzerland) and counted (2 min per probe) with a gamma-counter (LKB Clinigamma, Pharmacia AG, Dübendorf, Switzerland). All tests were done in quadruplicate. Specific tumour cell lysis was calculated according to the formula:

$$(\text{experimental c.p.m.} - \text{spont. c.p.m.}) / (\text{max. c.p.m.} - \text{spont. c.p.m.}) \times 100$$

Lytic units (LU) per ml blood were calculated based on the E/T ratio at the intercept of 20% specific lysis of 5,000 target cells.

### Results

Eight patients were treated with at least three full cycles of IL-2 and INF α-2a. None of the patients had a history of endocrine disorders. All had normal thyroid function tests and normal levels of anti-thyroglobuline and anti-microsomal antibodies before treatment was started. Patient characteristics, response to treatment, and results of thyroid function studies are summarised in Table I. Four patients had normal thyroid function during and after completion of treatment. Four patients developed thyroid dysfunction.

The evolution of thyroid dysfunction followed a similar pattern in all four patients with laboratory evidence of hyperthyroidism during the second or third cycle of treatment in two patients each, followed by laboratory evidence of hypothyroidism within 2 weeks. The changes in TSH and T4 levels over time for patient 3/M 1944, which are representative of all four patients with thyroid dysfunction, are depicted in Figure 1.

In patients 1 and 2 there was no clinical evidence for thyroid dysfunction and thyroid function studies returned to normal within 10–22 weeks after discontinuation of IL-2/INF α-2a therapy. Patients 3 and 4 became clinically symptomatic with tachycardia of up to 150 beats $min^{-1}$ requiring therapy with a β-blocker. This was followed by an inappropriate degree of fatigue in both and the development of a unilateral carpal tunnel syndrome in patient 4. Both were substituted with sodium L-thyroxine 0.5–1.0 mg daily for 11 and 18 weeks.

In one patient with hypothyroidism anti-thyroglobuline antibodies became elevated from 32 IU $ml^{-1}$ at baseline to a maximum of 1,236 IU $ml^{-1}$ at week 5. They returned to normal within 6 months. In all other patients anti-thyroglobuline and anti-microsomal antibodies remained at normal values. No other autoantibodies, including anti-mitochondrial, anti-nuclear, anti-parietal cell or anti-beta islet cell antibodies were detected.

No patient had clinically detectable thyroid enlargement. Patients 2, 3 and 4 underwent fine-needle aspirations of the thyroid during the third or fourth cycle of treatment. All three had cytological evidence of chronic thyroiditis manifested in a mixture of lymphocytes, plasma cells, histiocytes and thyroid epithelial cells (Figure 2). The fine-needle aspirates of the three patients and of three untreated controls were examined for expression of class II antigens by immunocytochemistry. Thyroid epithelial cells of all three patients revealed strong expression of HLA-DR antigen (Figure 3a), while controls remained antigen negative (Figure 3b). Patient 1 had a subclinical course which was detected only later during treatment and therefore had no biopsy done.

Functional NK and LAK activity of PNMCs and the number of PMNCs with NK markers were determined at baseline and after stimulation at day 8 of the third

**Table I** Patient characteristics

| *Number sex date of birth* | *Tumour* | *Response after 4 cycles* | *TSHmax*[a] | *TSHmin*[a] | *T4max*[b] | *T4min*[b] | *Antithyroid antibodies*[c] | *FNA* | *L-Thyroxin replacement* |
|---|---|---|---|---|---|---|---|---|---|
| 1/M/1944 | RCC | PD | 22.7 | <0.08 | 165 | 64 | not elevated | no | no |
| 2/F/1940 | MM | MR | 2 | <0.08 | 197 | 56 | not elevated | yes | no |
| 3/M/1944 | MM | PD | 61.4 | <0.08 | 218 | 32 | 1236 IE $ml^{-1}$ | yes | yes |
| 4/F/1946 | MM | MR | 63 | <0.08 | 192 | 5 | not elevated | yes | yes |
| 5/F/1929 | MM | PD | 3.5 | 1.8 | 132 | 92 | not elevated | no | no |
| 6/M/1930 | RCC | NC | 1.2 | 0.4 | 147 | 70 | not elevated | no | no |
| 7/F/1940 | RCC | PD | 1.5 | 0.5 | 98 | 72 | not elevated | no | no |
| 8/M/1930 | RCC | PD | 2.5 | 1.6 | 102 | 62 | not elevated | no | no |

[a]Normal = 0.1–4 mU $l^{-1}$. [b]Normal = 50–150 nmol $l^{-1}$. [c]Normal = 0–350 mU $l^{-1}$.

**Figure 1** Serum thyroxine and thyrotropin concentration of patient 3/M/1944 with autoimmune thyroiditis in relation to treatment with IL-2 and INF α-2a.

**Figure 2** Papanicolaou stain of fine needle aspirate of the thyroid from patient 3/M/1944, showing chronic inflammation with lymphocytes, plasmacells and histiocytes. Original magnification × 330.

**Figure 3** **a**, Immunocytochemistry of fine needle aspirate of patient 3/M/1944 showing strong HLA-DR expression on thyroid epithelial cells. Original magnification × 528. **b**, Immunocytochemistry of fine needle aspirate of a normal thyroid as control, showing negative staining for HLA-DR. Original magnification × 528.

cycle of treatment. Mean baseline NK and LAK activity was 117 LU $ml^{-1}$ and 6.7 LU $ml^{-1}$, respectively and increased to 385 LU $ml^{-1}$ and 98 LU $ml^{-1}$, respectively on day 8 of cycle 3. Baseline CD16 positive cells were 0.25 g $l^{-1}$ and increased to 1.05 g $l^{-1}$ on day 8 of cycle 3. Figure 4 compares these immune modulatory parameters in patients with and without thyroid dysfunction. Patients with thyroid dysfunction tended to have a higher stimulated NK activity and LAK activity than euthyroid patients. The number of CD16 positive cells at baseline and after stimulation was significantly higher in patients with thyroid dysfunction. No significant differences were found in the absolute number of lymphocytes and the number of CD8, CD56 and CD57 positive cells.

### Discussion

Our observations suggest that transient thyroid dysfunction is a common finding with combined IL-2/INF α-2a therapy. In our series of eight and a similar series of seven patients from another center (communicated by Pichert et al., 1989) half of the patients treated for melanoma and renal cell carcinoma were found to have thyroid dysfunction, a much higher frequency than with IL-2 and LAK cell therapy, where it has been reported in seven of 34 (21%) patients treated for melanoma, renal cell carcinoma or colon carcinoma (Atkins et al., 1988), or with human leukocyte interferon therapy, where it has been reported in seven of 49 (14%) patients treated for carcinoid (Burman et al., 1986) and three of 13 (23%) patients treated for breast cancer (Fentimann et al., 1988).

The thyroid dysfunction observed in our study is caused by an autoimmune thyroiditis, as evidenced by the evolution of thyroid function studies, the cellular infiltrates in fine-needle aspirates and by the expression of HLA class II antigens by thyroid epithelial cells. This condition evolved within 4–6 weeks of alternate weekly IL-2/INF α-2a therapy with first a hyperthyroid phase of 2 weeks duration followed by a hypothyroid phase lasting up to 24 weeks. Three patients had a fine-needle aspirate of the thyroid during the hypothyroid state and all three showed a pattern consistent with an autoimmune thyroiditis with a mixed lymphocytic/histiocytic infiltrate and strong staining of thyrocytes for HLA class II antigens. In contrast to Hashimoto's thyroiditis which is usually long lasting and often evolves into a permanent hypothyroid state (Doniach et al., 1979), the thyroiditis observed in our patients was self-limited.

Bottazzo et al. (1983) initially suggested aberrant HLA class II expression on thyrocytes as a key factor for the induction of autoimmune thyroiditis. Normal thyrocytes do not express HLA class II antigens, whereas strong expression has been demonstrated in patients with autoimmune thyroiditis (Hanafusa et al., 1983). *In vitro*, HLA class II expression can be induced on cultured human thyrocytes by adding activated T-cells or more directly recombinant INF γ (Todd et al., 1985). TNF α enhances the effect of INF γ (Buscema et al., 1989); recombinant IL-2 (Todd et al., 1985) or INF α (Burman et al., 1986) added in the absence of INF γ fail to induce HLA class II on human thyrocytes.

**Figure 4** Immunomodulatory effect of IL-2/INF α-2a in four patients with thyroiditis (●) and four patients without thyroiditis (○). Mean values and standard deviation at study entry and at day 8 of cycle 3. **a**, NK activity of PMNCs against K562 target cells. **b**, LAK activity of PMNCs against Daudi target cells. **c**, Circulating CD8-positive cells. **d**, Circulating CD16-positive cells.

In thyroid glands of patients with Hashimoto's thyroiditis or Graves' disease the highest epithelial expression of HLA-DR antigen was found in areas of most intense lymphoid infiltrates (Flynn *et al.*, 1988). Therefore it is possible that HLA-DR expression is induced as a result of intercellular communication of activated lymphocytes and macrophages with thyroid epithelial cells; a process which involves cytokines and which results in an inappropriate antigen recognition of thyrocytes by autoagressive T cell clones. In addition to INF γ and TNF α other cytokines may be involved such as interleukin-1 which has been found to affect directly the function of thyrocytes *in vitro* (Rasmussen *et al.*, 1987).

With the exception of two case reports treated with IL-2 (van Liessum *et al.*, 1989; Hartmann *et al.*, 1989), hypothyroidism has not been observed with the use of IL-2 or INF α-2a alone. We suggest that the high frequency of thyroiditis observed with IL-2/INF α-2a treatment is based on several factors. (1) The combined treatment with IL-2 and INF α-2a may lead to a pronounced secretion of secondary cytokines including INF γ, TNF α and IL-1 (Gemlo *et al.*, 1988; del Prete *et al.*, 1987). Whereas INF γ and TNF α have also been detected in the serum of patients after IL-2 alone the titers are more impressive after treatment with IL-2 and LAK cells (Gemlo *et al.*, 1988), which comprise well established sources of cytokines such as IL-1, TNF α and INF γ. (2) The development of thyroiditis may also depend on a more pronounced recruitment of autoreactive T cell clones or a hyperinduction of the cytolytic potential of mononuclear cells by IL-2 and INF α and not with either agent alone. In the mouse model it has been shown, that the frequency of lytic effector cells in the liver is significantly greater with the combination than with IL-2 or INF α-2a alone (Brunda *et al.*, 1986). Furthermore, in patients with Hashimoto's thyroiditis the proportion of INF γ producing T cell clones derived from thyroid infiltrates was significantly higher than in the peripheral blood of healthy donors and there was a positive relationship between high INF γ production and NK activity of T cell clones of peripheral blood and thyroid glands of patients with Hashimoto's disease (del Prete *et al.*, 1987). In this regard it is of note that in our small series patients with thyroiditis tended to have a higher lytic potential of peripheral blood mononuclear cells and a significantly higher number of CD16 positive PMNCs than patients without thyroiditis.

It is pertinent to ask why autoimmune disease has been restricted to the thyroid and why only selected patients were afflicted. In general there appears to be a genetic basis for the tendency to develop autoimmunity as evidenced by HLA associations and family studies which are also indicative for a preference for a particular organ (Flynn *et al.*, 1988). In experimental models a restricted usage of some T cell receptor Vβ genes in the T cell response to defined determinants on autoantigens had been shown (Zamvil *et al.*, 1988). An individual disease susceptibility has been suggested by the observation of genetic differences in the amount of MHC class II expression following IFN γ treatment of parenchymal cells (Massa *et al.*, 1987).

HLA class II antigen expression on tumour cells has been found associated with responsiveness to IL-2 and LAK cell therapy and an association between hypothyroidism and tumour response to IL-2 and LAK cells has been suggested (Cohen *et al.*, 1987). This hypothesis is also supported by our preliminary observations together with a group of investigators in Dublin with four tumour responses to IL-2/INF α-2a treatment (two minor and two partial) in eight patients with hypothyroidism, and no responses in seven euthyroid patients (communicated by Pichert *et al.*, 1989). Together these observations suggest that the expression of HLA class II antigens and auto-reactive cytotoxic T-lymphocytes might contribute to the development of thyroiditis as well as to the anti-tumour response, in addition to NK and LAK cells which act independent of the HLA class II system.

Self-limited thyroiditis with hyperthyroidism followed by hypothyroidism occurs in half of the patients treated with IL-2 and INF α-2a. Clinical or laboratory thyroid dysfunction should be anticipated in future clinical trials with these substances. Also the possibility of a positive correlation between tumour response and the occurrence of thyroiditis needs further evaluation.

We thank Prof. A. Fontana for helpful discussion.

### References

ATKINS, M.B., MIER, J.W., PARKINSON, D.R. & 4 others (1988). Hypothyroidism after treatment with interleukin-2 and lymphokine-activated killer cells. *N. Engl. J. Med.*, **318**, 1557.

BOTTAZZO, G.F., PUJOL-BORRELL, R. & HANAFUSA, T. (1983). Role of aberrant HLA-DR expression and antigen presentation in induction of endocrine autoimmunity. *Lancet*, **ii**, 1115.

BRUNDA, M.J., TARNOWSKI, D. & DAVATELIS, V. (1986). Effect of combinations of recombinant interferon alpha and interleukin-2 on tumor metastases and cytotoxic cell function. In *Natural Immunity, Cancer and Biological Response Modification*, Lotzova & Herbermann (eds) p. 235. Karger: Basel.

BURMAN, P., TÖTTERMAN, T.H., ÖBERG, K. & KARLSSON, F.A. (1986). Thyroid autoimmunity in patients on long term therapy with leucocyte-derived interferon. *J. Clin. Endocrinol. Metab.*, **63**, 1086.

BUSCEMA, M., TODD, I., DEUSS, U. & 4 others (1989). Influence of tumor necrosis factor-alpha on the modulation by interferon-gamma of HLA class II molecules in human thyroid cells and its effect on interferon-gamma binding. *J. Clin. Endocrinol. Metab.*, **69**, 433.

COHEN, P.J., LOTZE, M.T., ROBERTS, J.R., ROSENBERG, S.A. & JAFFE, E.S. (1987). The immunopathology of sequential tumor biopsies in patients with Interleukin-2: correlation of response with T-cell infiltration and HLA-DR expression. *Am. J. Pathol.*, **129**, 208.

DEL PRETE, G.F., TIRI, A., MARIOTTI, S., PINCHERA, A., RICCI, M. & ROMAGNANI, S. (1987). Enhanced production of gamma-interferon by thyroid-derived T cell clones from patients with Hashimoto's thyroiditis. *Clin. Exp. Immunol.*, **69**, 323.

DONIACH, D., BOTTAZZO, G.F. & RUSSELL, R.C.G. (1979). Goitrous autoimmune thyroiditis (Hashimoto's disease). *J. Clin. Endocrinol. Metab.*, **8**, 63.

DUTCHER, J.P., CREEKMORE, S., WEISS, G.R. & 11 others (1989). A phase II study of interleukin-2 and lymphokine-activated killer cells in patients with metastatic melanoma. *J. Clin. Oncol.*, **7**, 477.

FENTIMANN, I.S., BALKWILL, F.R., THOMAS, B.S., RUSSELL, M.J., TODD, I. & BOTTAZZO, G.F. (1988). An autoimmune aetiology for hypothyroidism following interferon therapy for breast cancer. *Eur. J. Cancer. Clin. Oncol.*, **24**, 1299.

FISHER, R.I., COTMAN, C.A. Jr, DOOSHOW, J.H. & 11 others (1988). Metastatic renal cancer treated with interleukin-2 and lymphokine activated killer cells. A phase II clinical trial. *Ann. Intern. Med.*, **108**, 518.

FLYNN, S.D., NISHIYAMA, R.H. & BIGOS, T.S. (1988). Autoimmune thyroid disease: immunological, pathological, and clinical aspects. *Crit. Rev. Clin. Lab. Sci.*, **26**, 43.

GEMLO, B.T., PALLADINO, M.A. Jr, JAFFE, H.S., ESPEVIK, T.P. & RAYNER, A.A. (1988). Circulating cytokines in patients with metastatic cancer treated with recombinant interleukin 2 and lymphokine-activated killer cells. *Cancer Res.*, **48**, 5864.

HANAFUSA, T., CHIOVATO, L., DONIACH, D., PUJOL-BORELL, R., RUSSELL, R.C.G. & BOTTAZZO, G.F. (1983). Aberrant expression of HLA-DR antigen on thyrocytes in Grave's disease: relevance for autoimmunity. *Lancet*, **ii**, 1111.

HARTMANN, L.C., URBA, W.J., STEIS, R.G. & 4 others (1989). Hypothyroidism after interleukin-2 therapy (letter). *J. Clin. Oncol.*, **7**, 686.

MASSA, P.T., TER MEULEN, V. & FONTANA, A. (1987). Hyperinducibility of Ia antigen on astrocytes correlates with strain-specific susceptibility to experimental encephalomyelitis. *Proc. Natl Acad. Sci. USA*, **844**, 4219.

PICHERT, G., JOST, L.M., OELZ, O. & 8 others (1989). Combination of interleukin-2 and interferon alpha-2a in advanced renal cell carcinoma and melanoma: preliminary results and patient tolerance. *ECCO*, **5**, 717.

RASMUSSEN, A.K., BECH, K., FELDT-RASMUSSEN, U. & 4 others (1987). The influence of interleukin-1 on the function of in vitro cultured human thyroid cells in monolayer. *Acta Endocrinol. Suppl.*, **281**, 93.

ROSENBERG, S.A., LOTZE, M.T., MUUL, I.M. & 10 others (1987). A progress report on the treatment of 157 patients with advanced cancer using lymphokine-activated killer cells and interleukin-2 or high dose interleukin-2 alone. *N. Engl. J. Med.*, **316**, 889.

STAHEL, R.A., SCULIER, J.P., JOST, L.M. & 7 others (1989). Tolerance and effectiveness of recombinant interleukin-2 (r-met Hu Il-2 [ala-125]) and lymphokine-activated killer cells in patients with metastatic solid tumor. *Eur. J. Cancer Oncol.*, **25**, 965.

TODD, I., PUJOLL-BORELL, R., HAMMOND, L.J., BOTTAZZO, G.F. & FELDMANN, M. (1985). Interferon-gamma induces HLA-DR expression by thyroid epithelium. *Clin. Exp. Immunol.*, **61**, 265.

VAN LIESSUM, P.A., DE MULDER, P.H.M., MATTIJSSEN, E.J.M., CORSTENS, F.H.M. & WAGENER, D.J.TH. (1989). Hypothyroidism and goitre during interleukin-2 therapy without LAK cells. *Lancet*, **i**, 234.

ZAMVIL, S.S., MITCHELL, D.J., LEE, N.E. & 7 others (1988). Predominant expression of a T cell receptor V $\beta$ gene subfamily in autoimmune encephalomyelitis. *J. Exp. Med.*, **167**, 1586.